# New Appliances and Things Medical

**Published:** 1903-06-27

**Authors:** 


### NEW APPLIANCES AND THINGS MEDICAL.

[We shall be glad to receive, at our Office, 28 & 29 Southampton Street Strand, London, W.C., from the manufacturers, specimens of all new preparations and appliances which may be brought out from time to time.]

### "ZYMINE" PEPTONISING TUBES.

(FAIRCHILD BROTHERS AND FOSTER, NEW YORK. LONDON AGENTS: BURROUGHS, WELLCOME AND CO., SNOW HILL, LONDON, E.C.)

THE popular method of peptonising milk and other liquid foods, known as the "Fairchild," has recently been rendered more practical by the publication of a list of recipes in book form, which are so arranged that they are immediately accessible for reference. The "Zymine" or peptonising powders are packed in small glass tubes hermetically sealed by specially prepared corks. The method is so simple, rapid, and satisfactory that it is likely to supersede all others.

